# Ultra‐High Pressure Phosphor Based on an Eco‐Friendly Perovskite Via Isovalent and Heterovalent Co‐Doping Engineering

**DOI:** 10.1002/advs.202515142

**Published:** 2025-11-14

**Authors:** Xinyu He, Tao Xiong, Xiyao Wang, Yongchang Han, Qingqin Ge, Nannan Shi, Liwei Jiang, Cheng Sun, Yinan Zhang, Hengnan Liang

**Affiliations:** ^1^ College of Physical Science and Technology Dalian University Dalian 116622 China; ^2^ School of Physics Dalian University of Technology Dalian 116024 China; ^3^ College of Science Beihua University Jilin 132013 China; ^4^ Thermo Fisher Scientific China Shanghai 201203 China; ^5^ Key Laboratory of Physics and Technology for Advanced Batteries (Ministry of Education) College of Physics Jilin University Changchun 130012 China; ^6^ College of Physics Jilin University Changchun 130012 China

**Keywords:** heterovalent doping, photoluminescence, tin halide perovskite, ultra‐high pressure

## Abstract

Tin halide perovskites are good eco‐friendly alternatives to lead halide perovskites, whose toxicity problem can effectively be eliminated. In order to improve the fluorescence properties, in this work, a novel non‐toxic perovskite phosphor is successfully synthesized, employing both isovalent and heterovalent co‐doping chemical engineering. The photoluminescence of this new phosphor is prominent in the visible light regime, and the corresponding color can readily be tuned from green to red. Based on DFT calculations and time‐resolved photoluminescence spectra, a physical model that accounts for the light emission is proposed, where efficient energy transfers between self‐trapped excitons are addressed. Interestingly, the physical effect of Sb^3 +^ heterovalent doping on the phosphor is observed in transient absorption spectra, manifesting itself as a spectral broadening. Remarkably, with increasing the pressure on the phosphor from 1 atm up to an ultra‐high value of 23.5 GPa, an interesting evolution of the photoluminescence spectrum is clearly revealed. This new phosphor and the dual‐ion co‐doping strategy may advance sustainable material science.

## Graphical abstract

A non‐toxic tin halide perovskite phosphor is synthesized via co‐doping chemical engineering. The photoluminescence of the phosphor can be tuned from green to red. An interesting evolution of fluorescence is revealed, as the pressure on the phosphor is first compressed from 1 atm up to an ultra‐high value of 23.5 GPa, and is then gradually released.

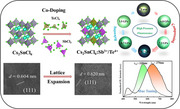

## 1 Introduction

Thanks to the extraordinary properties, including unique crystal structures, excellent light absorption, high charge carrier mobility, good bandgap tunability, high power conversion efficiency (PCE), as well as great light emission, lead halide perovskites (LHPs) are important materials,^[^ ^1^, ^2^, ^3^, ^4^, ^5^, ^6^, ^7^, ^8^, ^9^, ^10^ ^]^ with many successful optoelectronic applications,^[^ ^11^ ^]^ such as solar cells,^[^ ^12^, ^13^ ^]^ photodetectors,^[^ ^14^, ^15^, ^16^ ^]^ light emitting diodes (LEDs),^[^ ^17^, ^18^, ^19^, ^20^, ^21^, ^22^ ^]^ and lasers,^[^ ^23^ ^]^ etc. However, there is a lethal problem for LHPs, that is, lead is toxic. Recently, tin‐based perovskites have been intensively studied, and are deemed as promising alternatives.^[^ ^24^, ^25^, ^26^, ^27^, ^28^ ^]^ Compared to LHPs' great characteristics, several aspects of tin‐based perovskites still need be solved and improved, including the oxidation of Sn^2 +^, the high density of defects, the low PCE, and the instability of the perovskite, etc. Amongst various strategies, the ion doping has been considered as a convenient and useful method, to improve the physical performance of Sn‐based perovskites.^[^ ^29^, ^30^, ^31^, ^32^ ^]^ Generally, in pristine Cs_2_SnCl_6_ crystals (one stable tin‐based perovskite) the photoluminescence (PL) is attributed to its intrinsic self‐trapped exciton (STE).^[^ ^33^ ^]^ Upon doping, the newly‐introduced dopant may give rise to additional extrinsic self‐trapped exciton. Differing from the blue light emission of pristine Cs_2_SnCl_6_, the energy transfer between STEs in doped perovskites governs the overall emission spectrum, resulting in various hues of the light.^[^ ^34^ ^]^ Therefore, the introduction of suitable STEs into pristine perovskites, and the proper manipulation of the energy transfer between STEs are the key to the synthesis engineering of tin‐based perovskites with desirable luminescence properties.

Heterovalent doping is believed to be an efficient strategy to fulfil various luminescence properties of the Cs_2_SnCl_6_ perovskite, and it has been shown that the formation of Cl vacancy defects that are induced by the heterovalent dopants are responsible for their emissions. For example, a highly efficient deep‐blue emission with a high PL quantum yield (PLQY) was found in Bi^3 +^ doped Cs_2_SnCl_6._^[^ ^33^ ^]^ A red shift of the PL peaks from 455 to 483 nm was presented in Cs_2_SnCl_6_:Bi^3 +^ via a post‐annealing treatment.^[^ ^35^ ^]^ Besides, an enhancive Jahn‐Teller distortion in the excited state and the resulting intense broadband blue photoluminescence at 450 nm was also addressed in 0D structure of Cs_2_SnCl_6_:Bi^3 +^.^[^ ^36^ ^]^ In addition, white light was achieved by dual‐STEs emissions with blue and yellow colors in Cs_2_SnCl_6_:La^3 +^ microcrystals.^[^ ^34^ ^]^ Recently, a broad PL emission centered at 550 nm was also observed in Cs_2_SnCl_6_:Y^3 +^, demonstrating its potential as a white light phosphor.^[^ ^37^ ^]^

In addition to the atmospheric pressure environment, the luminescence properties of halide perovskites may also be varied by high pressures; pressure‐induced emission (PIE) has been extensively studied, and several advances have been achieved. For instance, it was reported that the initially non‐fluorescent perovskite nanocrystals of Cs_4_PbBr_6_ could show a distinct emission under a high pressure of 3.01 GPa.^[^ ^38^ ^]^ The enhanced emission of a halide perovskite of (C_5_H_7_N_2_)_2_ZnBr_4_ was addressed via pressure treatment, where the pressure processing could also yield the tuning of “sky blue light” before compression to “cool daylight”^[^ ^39^ ^]^. Recently, a dramatic fluorescence enhancement was also found on an yttrium‐doped perovskite of Cs_2_SnCl_6_ under high pressures.^[^ ^37^ ^]^

Considering the +4 valence state of Sn in the perovskite of Cs_2_SnCl_6_, isovalent Te^4 +^ ions and heterovalent Sb^3 +^ ions are both used as dopants, in order to pursue innovative properties. In this work, the successful synthesis of the non‐toxic perovskite (i.e., Cs_2_SnCl_6_:Sb^3 +^/Te^4 +^) is reported for the first time. Based on its photoluminescence, an efficient color tuning from green to red is fulfilled. Associated energy models are proposed, with which the energy transfer between STEs are addressed. In addition to the atmospheric pressure, the new phosphor is also compressed under an external pressure as great as 23.5 GPa, and an interesting fluorescence evolution is clearly revealed; the effects of the heterovalent doping is discussed, with the aid of Raman spectra under pressure.

## 2 Synthesis and Characterizations

All samples were synthesized by a one‐pot solvothermal method (Figure S1, Supporting Information). Cesium chloride, tin chloride, antimony chloride, and tellurium chloride were added to HCl aqueous solution in an autoclave. It was heated at 180°C for 10 h to produce a saturated solution, and then nucleation and growth of particles were induced while it was slowly cooled down from 180°C to room temperature for 30 h (for details, see the Supporting Information).

**Figure 1:**
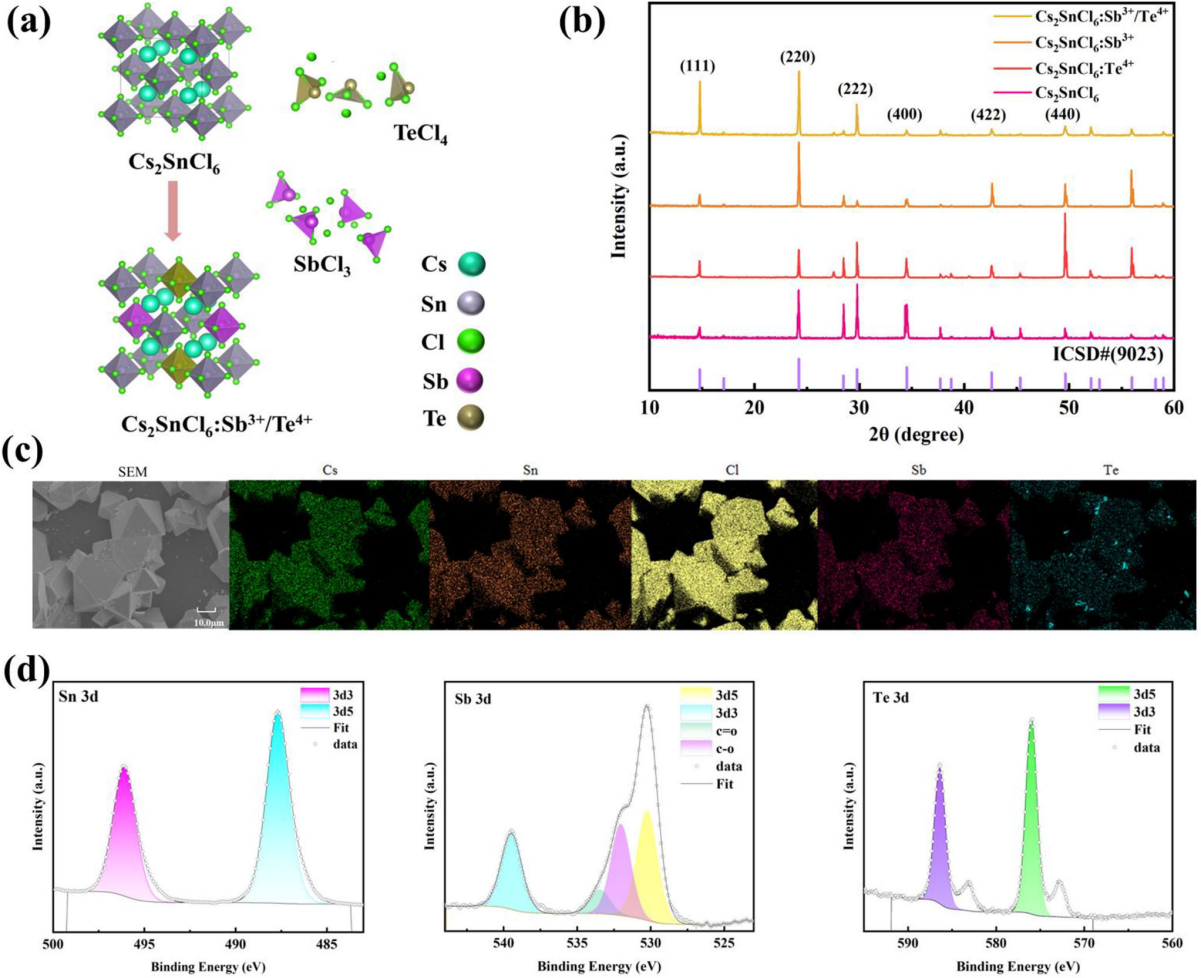
a) Schematic crystal structure of the perovskite of Cs_2_SnCl_6_, and Cs_2_SnCl_6_:Sb^3 +^/Te^4 +^. b) XRD patterns of Cs_2_SnCl_6_ with different dopants. (c) SEM image and corresponding EDS mappings of Cs, Sn, Cl, Sb, and Te for Cs_2_SnCl_6_:Sb^3 +^/Te^4 +^. d) High‐resolution XPS spectra in Sn 3d, Sb 3d, and Te 3d regions of Cs_2_SnCl_6_:Sb^3 +^/Te^4 +^.

The crystal structure of the perovskite is sketched in **Figure** **1**a. Each [SnCl_6_]^2 −^ octahedron is isolated, and Cs^+^ occupies the external cavities in the octahedra. Sb^3 +^ and Te^4 +^ dopants are supposed to substitute for the Sn^4 +^ sites in the centers of the octahedra. Referring to the XRD patterns of the samples (Figure 1b), all diffraction peaks are observed to be consistent with the Cs_2_SnCl_6_ crystal structure. In addition, they are also in good agreement with the simulated pattern for Cs_2_SnCl_6_ at bottom (ICSD #9023) with the $\mathrm{Fm}\bar{3}m$ space group. The XRD results reassure us that Sb and Te exist as minority, and the introduction of the Sb and Te dopants would not vary the crystal structure of Cs_2_SnCl_6_.

Based on the SEM image (Figure 1c), all elements are uniformly distributed and perfectly overlapped in the mapping diagram, indicating the successful doping of Sb and Te elements. The EDS analysis (Table S1, Supporting Information) suggests that the molar ratio of Cs:Sn:Cl is approximately 2:1:6, which is concordant with the target stoichiometry of the host lattice Cs_2_SnCl_6_. In Table S1 (Supporting Information), the atomic percentages for the elements across five different grains are also compared, and the corresponding elemental distribution histograms across the grains are plotted in Figure S2 (Supporting Information), illustrating that the distributions of the dopants are relatively uniform across multiple grains to some extent.

To further verify the successful incorporation of the dopants into the host lattice, transmission electron microscopy (TEM) images and high resolution TEM (HRTEM) patterns of pristine Cs_2_SnCl_6_ and co‐doped Cs_2_SnCl_6_:Sb^3 +^/Te^4 +^ are both presented in Figure S3 (Supporting Information). Given the fact that the ionic radii of Sn^4 +^, Sb^3 +^, and Te^4 +^ are 0.69, 0.76, and 0.97 Å, respectively, the incorporation of the dopants would result in a lattice expansion. This effect is clearly demonstrated by the experimental result that is indicated in Figure S3 (Supporting Information) where the *d*‐spacing of (111) plane of the co‐doped sample (i.e., 0.620 nm) is larger than that of the pristine one (i.e., 0.604 nm). Similar values and effects were also reported in Ref. [30], in which the dopants of Bi^3 +^ and Te^4 +^ were successfully co‐doped into the host lattice of Cs_2_SnCl_6_.

The valence states of the elements are determined by the XPS measurements (Figure 1d). The binding energy peaks at 487.69 and 496.20 eV are attributed to the 3d orbital of the Sn^4 +^ cation, verifying the +4 oxidation state of Sn (Ref. [34]). In Figure 1d, the valence states of Sb^3 +^ (i.e., 530.22 eV and 539.50 eV) and Te^4 +^ (576.00 and 586.40 eV) are also evident. To further probe the synergy effect of the Sb and Te co‐doping, the XPS experiments were also carried out on single‐element doping perovskites (i.e., Cs_2_SnCl_6_:Sb^3 +^, and Cs_2_SnCl_6_:Te^4 +^), respectively. The corresponding binding energies are listed in Table S2 (Supporting Information). Compared to single doping, the co‐doped sample has lower binding energies for both Sb and Te by 0.35 and 0.52 eV, respectively. This infers that upon co‐doping, the densities of the electron cloud surrounding Sb and Te are both increased, which may result in an enhancement in the luminescence intensity via the increase of the electron‐hole recombination probability.

## 3 Luminescence Physics and Energy Models

**Figure 2:**
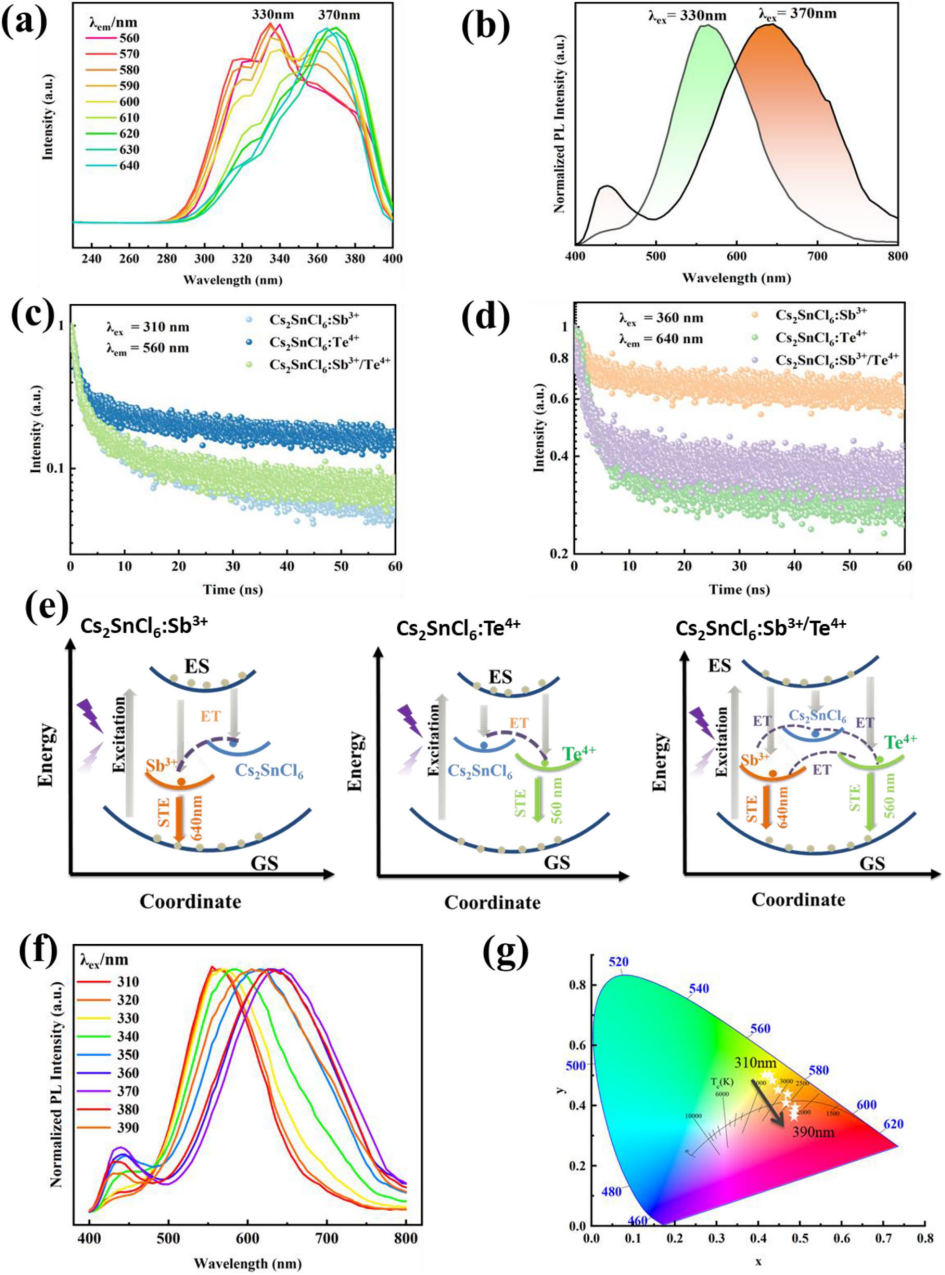
a) PLE and b) PL spectra of Cs_2_SnCl_6_:Sb^3 +^/Te^4 +^. c,d) TRPL decay curves of Cs_2_SnCl_6_:Sb^3 +^, Cs_2_SnCl_6_:Te^4 +^, and Cs_2_SnCl_6_:Sb^3 +^/Te^4 +^. e) Schematic energy diagram for Cs_2_SnCl_6_ with different dopants. f) PL spectra of Cs_2_SnCl_6_:Sb^3 +^/Te^4 +^, with a variety of excitation wavelengths. g) CIE color coordinates corresponding to the PL spectra in (f).

It is known that pristine Cs_2_SnCl_6_ crystals emit a blue PL (centered at about 440 nm) under a 350 nm excitation light, and the PL is attributed to the intrinsic STE emission that arises from the strong Jahn‐Teller distortion of [SnCl_6_]^2 −^ octahedra.^[^ ^34^ ^]^ The formation of STEs, correlated with localized charge distribution and strong carrier‐phonon coupling, is one advantage of inorganic perovskites that may result in fabulous optoelectronic properties. ^[^ ^40^, ^41^, ^42^ ^]^ In this work, the photoluminescence excitation (PLE) and PL spectra of the co‐doping perovskite are presented in **Figure** **2**a,b, respectively. Two distinct PLE peaks are revealed at 330 and 370 nm in Figure 2a. In Figure 2b, under the respective excitation of 330 and 370 nm, two broad emissions centered at different wavelengths are also observed, with a large overlap around 600 nm. It is suspected that these two PL emissions may originate from different dopants, and the discrimination of the peaks in PL spectrum is done with the aid of the PL and PLE spectra on single‐doping samples (Figure S4, Supporting Information). It is clear that Cs_2_SnCl_6_:Sb^3 +^ emits a red PL centered at 640 nm with a PLE peak at 370 nm (Figure S4a,b, Supporting Information), while a green light at 560 nm is for Cs_2_SnCl_6_:Te^4 +^ with a PLE peak at 330 nm. Referring back to Figure 2b, two PL emissions are attributed to the introduction of Sb and Te, respectively. Specifically, the 640 nm PL is ascribed to the extrinsic STE that is induced by the formation of [SbCl_5_]^2 −^ polyhedron, and the 560 nm PL is correlated to the STE that arises from the octahedron of [TeCl_6_]^2 −^. Similar observations and mechanisms were also reported in Ref.[34], where La^3 +^ was doped in Cs_2_SnCl_6_. In Figure 2b, more importantly, the overlapping between the PL spectra indicates a potential energy transfer between two STEs, and it is this large overlap that can give rise to great hue tunability of the co‐doped perovskite. Besides, the PLQY of the Cs_2_SnCl_6_:Sb^3 +^/Te^4 +^ phosphor was measured to 12.1 ± 0.6.

The luminescence dynamics is probed by the time‐resolved PL (TRPL) experiments. Under the excitation of both 310 and 360 nm lasers, the time‐dependent decay curves were measured at 560 and 640 nm, respectively (Figures 2c,d). All curves are well fitted to a bi‐exponential function, and the corresponding values for the lifetimes are tabulated in Table S3 (Supporting Information). The fast lifetime (τ_α_) of the 310 nm excitation, ranging between 1.11 and 1.18 ns, is almost the same for all samples, and it is also the same as the pristine Cs_2_SnCl_6_ (i.e., 1.02 ns^[^ ^34^ ^]^). This observation infers that τ_α_ belongs to the intrinsic STE from the pristine Cs_2_SnCl_6_. Interestingly, for both 310 and 360 nm lasers, the slow lifetime (τ_β_) of the co‐doped sample is shorter than both single‐doping ones. For example, τ_β_ is 23.03 ns for Cs_2_SnCl_6_:Sb^3 +^/Te^4 +^ under 360 nm, which is shorter than 36.05 or 43.23 ns. This phenomenon can well be accounted for by the aforementioned STE emission mechanism: with two extrinsic STEs, one more relaxation pathway is created in the co‐doped perovskite, compared with single‐doping ones; this results in a faster relaxation of the excited population with the aid of efficient energy transfer between STEs, manifesting itself as a shorter lifetime.

Based on both PL spectra and TRPL curves, a schematic energy diagram is proposed in Figure 2e, where the Sb and Te dopants can induce the formation of individual STEs. In the co‐doped sample, the 640 nm PL is dominated by the Sb dopants, and the 560 nm PL is with Te. Besides, the large overlap in PL spectra and the faster relaxation both indicate efficient energy transfers between these STE states. The model is further supported by the DFT calculations (Figure S5, Supporting Information), where the bandgaps and the density of states (DOS) are plotted. The computed bandgap of Cs_2_SnCl_6_:Sb^3 +^/Te^4 +^ is between that of single‐doping ones, indicating the good bandgap tunability that benefits from the co‐doping engineering strategy.

Different from single‐doping samples whose hue cannot be changed by the excitation light (Figure S4, Supporting Information), according to above physical mechanism, it is possible that the hue of the co‐doped phosphor can be varied, via changing the ratio of STE emissions through the efficient energy transfer. This is fulfilled in our phosphor of Cs_2_SnCl_6_:Sb^3 +^/Te^4 +^, by changing the excitation wavelength (Figure 2f). It is significant that the PL spectrum is gradually moved toward larger wavelength regime as the excitation wavelength is raised. The hue is systematically tuned and its corresponding CIE coordinates are given in Figure 2g.

**Figure 3:**
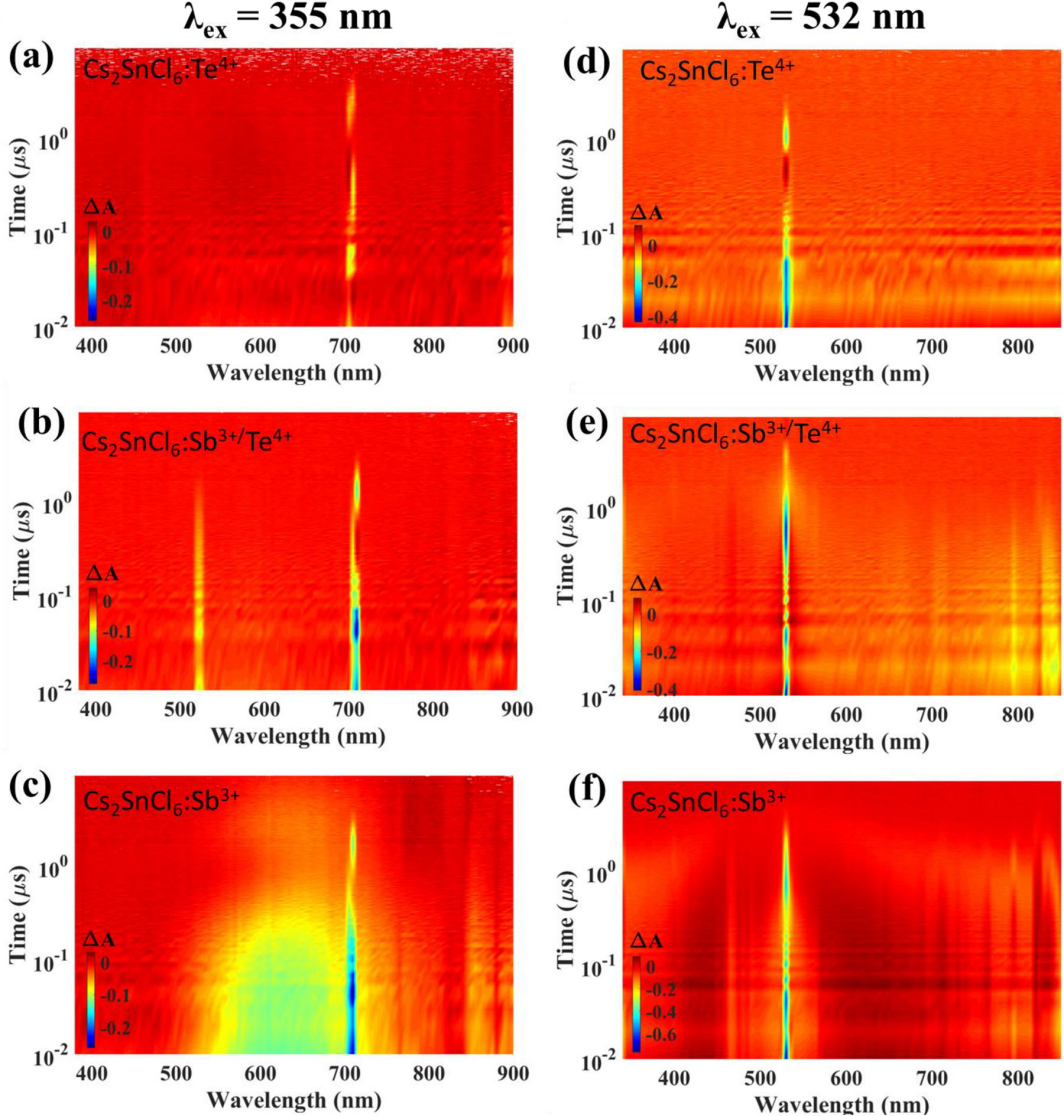
a–c) Contour plot of TAS of Cs_2_SnCl_6_ with different dopants, upon photoexcitation at 355 nm. d–f) Contour plot of TAS of Cs_2_SnCl_6_ with different dopants, upon photoexcitation at 532 nm.

To further probe the dynamics, the transient absorption spectra (TAS) were also performed on co‐doped and single‐doping perovskites (**Figure** **3**). Based on Figure 3, the pump‐induced absorption (PIA) signals (Δ*A*) as a function of probe wavelength or time are also plotted in Figure S6 (Supporting Information). Note that the signals at 532 nm through Figure 3d–f, and that at 710 nm through Figure 3a–c are the ground state bleaching (GSB) signals, which is induced by the photoexcitation laser with a wavelength of 532 and 355 nm, respectively. In Figure 3b the negative signal at 520 nm is attributed to the stimulated emission (SE) of the co‐doped sample, since this wavelength is normally longer than the excited wavelength (i.e., 355 nm in this case). In more details, before the SE process occurs, the photo‐excited atoms may lose certain energy due to the interaction with the lattice. Thus, the energy associated with the SE signal is usually smaller than the excitation energy, which corresponds to a longer wavelength of the probe light. In Figure 3c the appearance of SE spreading in a very broad region (510 – 750 nm) for the Sb‐doped sample is surprising. This indicates that in Cs_2_SnCl_6_:Sb^3 +^ the lower energy states to which the excited atoms relax are quite a broad band.

Based on the TAS results, the cooresponding energy diagram for the TAS dynamics of Cs_2_SnCl_6_:Sb^3 +^ is proposed (Figure S7, Supporting Information). Similarly, upon the excitation of 532 nm, the excited state absorption (ESA) signals (positive PIA signals in Figure 3f) are also very broad (400 – 850 nm), revealing a broad energy band for the excited state beyond the 532 nm level (i.e., 2.33 eV), as depicted in the dynamics diagram in Figure S7b (Supporting Information). The broadening of the energy levels in Cs_2_SnCl_6_:Sb^3 +^ may partially be attributed to the heterovalence effects, that is, trivalent Sb ions into a tetravalent Sn system. The associated lattice distortion is also supported by the DFT calculation for Sb‐doped crystals (Figure S8a, Supporting Information), where all bond lengths and bond angles present a deviation from the perfect cubic lattice (e.g., Te‐doped crystals in Figure S8b, Supporting Information). Referring back to Figure 3b, interestingly, the SE signal is quite narrow (520 nm) for the co‐doped sample, although the lattice distortion still exists as shown in Figure S8c (Supporting Information). Further, the DFT‐calculated crystal structure of Cs_2_SnCl_6_:Sb^3 +^ is also presented in Figure S9 (Supporting Information). It is clear that there are 6 Cl atoms that bond to the Sn atom to form the [SnCl_6_]^2 −^ octahedron, while only five Cl atoms are bonded to the Sb atom to form the [SbCl_5_]^2 −^ polyhedron.

Single‐component LEDs were fabricated based on the perovskites with different dopants (Figure S10a, Supporting Information). The synergy effect of the Sb and Te co‐doping is clearly shown: the Electroluminescence (EL) spectra of the co‐doped sample (centered at 600 nm) sits well in the middle of those of Sb‐doped (640 nm) and Te‐doped (560 nm) ones. The tuning effect of the hue is also solidly presented in terms of the corresponding CIE coordinates (Figure S10b, Supporting Information).

## 4 Pressure‐Dependent Fluorescence

**Figure 4:**
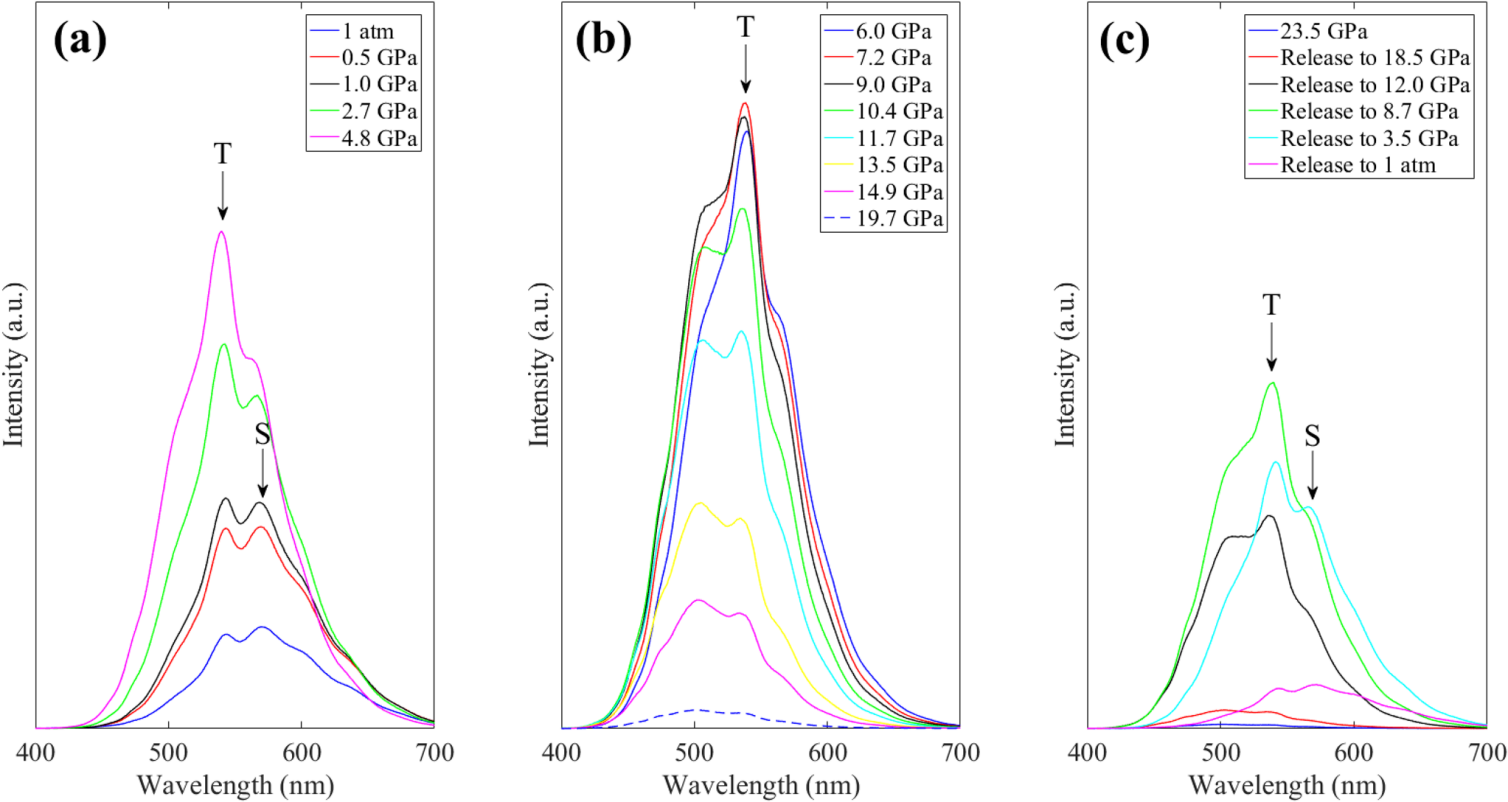
PL spectra of Cs_2_SnCl_6_:Sb^3 +^/Te^4 +^ under pressure. a) The pressure is increased from atmospheric pressure (1 atm) to 4.8 GPa. b) The pressure is further increased from 6.0 to 19.7 GPa. c) The pressure is gradually released from 23.5 GPa back to 1 atm. The peak at about 540 nm is indicated in ‘T’, and the one at around 580 nm is labeled ‘S’.

The fluorescence of the co‐doped phosphor was investigated under ultra‐high pressure (**Figure** **4**), where the pressure‐dependent fluorescence evolution is clearly revealed. By examining the overall PL spectrum, the intensity is first enhanced with increasing the pressure from the atmospheric pressure (1 atm) to 4.8 GPa (Figure 4a), and is then decreased dramatically as the pressure is further raised from 7.2 to 19.7 GPa (Figure 4b). In Figure 4c the PL intensity is increased again when the pressure is gradually released to 8.7 GPa; when the pressure is further released back to 1 atm, the intensity is then decreased. Besides, the evolution of the PL lineshape is more interesting, and the underlying pressure‐induced PL physics of the phosphor is manifested. Initially, two peaks are identified in the PL spectrum at 1 atm, and they are labeled ‘T’ and ‘S’, respectively, for brevity. Note that in this high‐pressure experiment, a laser source of 355 nm was used to photoexcite the phosphor, while in the steady state PL experiment (e.g., Figure 2b), a Xenon lamp with tunable wavelengths was utilized. Therefore, there are some variations in the PL spectra between the pressure‐dependent and the steady experiments. Referring back to Figure 4a, peak S is evident when the pressure is below 4.8 GPa. Regarding Figure 4b once the pressure is further raised above 4.8 GPa, peak S becomes negligible. In Figure 4c when the pressure on the phosphor is released below 3.5 GPa, peak S shows up again. The above observation implies that peak S is different from peak T, and the PL mechanism that is responsible for the emission of peak S would be quenched under a pressure that is greater than 4.8 GPa. In addition, By comparing the spectra between the ‘Release to 1 atm’ in Figure 4c and the ‘1 atm’ in Figure 4a, it is found that these two spectra are almost identical, except for the intensity. This indicates that the phosphor is reversible after compressing under an ultra‐high pressure as great as 23.5 GPa.

**Figure 5:**
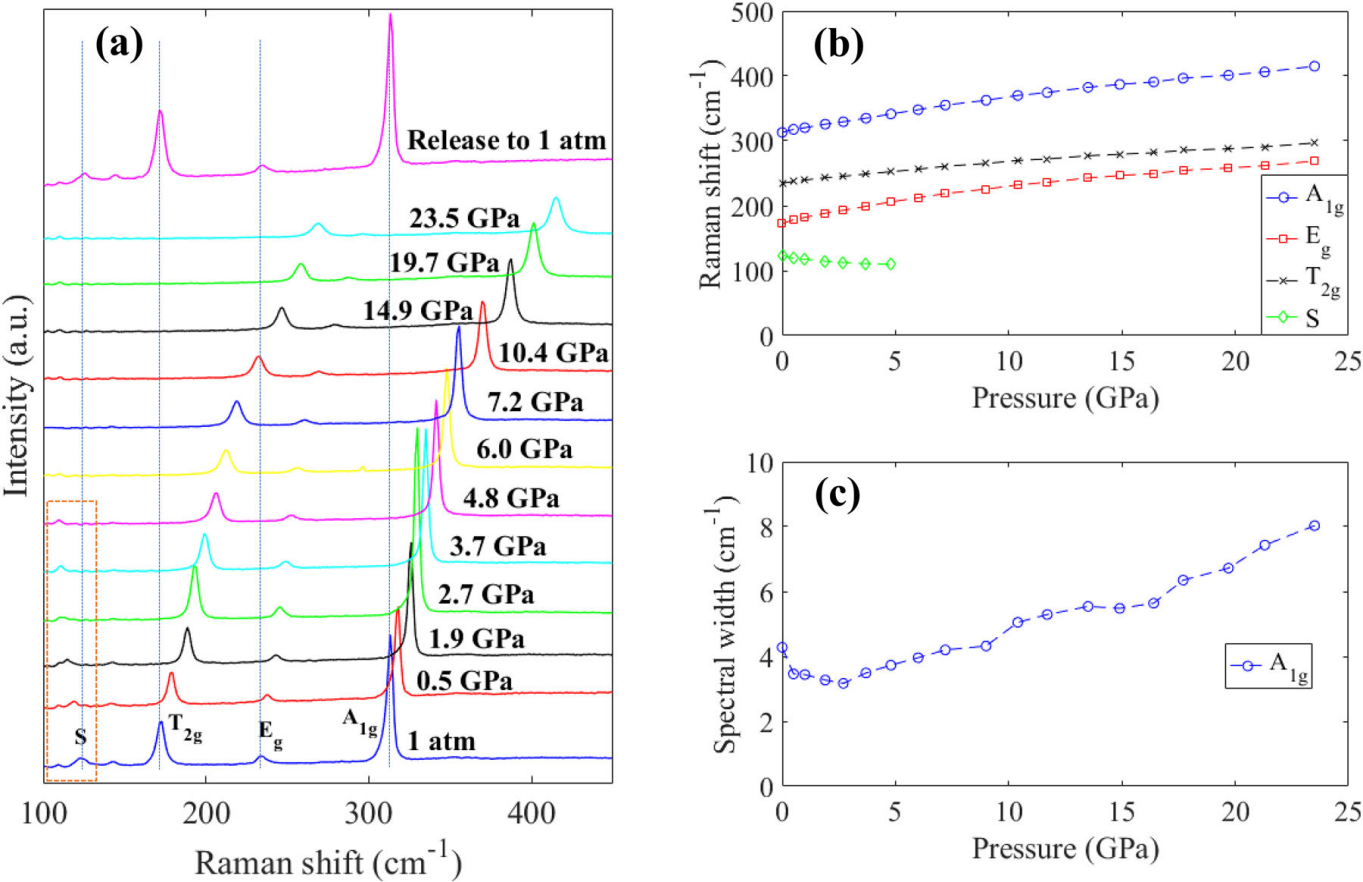
a) Raman spectra evolution of Cs_2_SnCl_6_:Sb^3 +^/Te^4 +^ under pressure. b) Raman peaks as a function of pressure. c) The spectral width of peak A_1*g*_ as a function of pressure.

To shed more light on the PL mechanism that governs the peak‐S emission, Raman spectra were also collected as a function of pressure, and the evolution is plotted in **Figure** **5**a. Regarding the spectrum at 1 atm, four peaks can be distinguished. The three ones at higher wavenumbers (labeled A_1*g*_, E_*g*_, and T_2*g*_, respectively) are identified to motions that are associated with the Cs_2_SnCl_6_ octahedra; peaks A_1*g*_ and E_*g*_ are correlated to the stretching vibrational motions of the octahedra, and peak T_2*g*_ is to do with the translational motion of the Cs atom, as well as the breathing mode of the relevant octahedron. Similar identifications have also been addressed in other halide perovskites in previous literatures^[^ ^43^, ^44^ ^]^. The relatively weak, yet evident Raman peak at about 120 cm^−1^ (labeled S) may be ascribed to vibrational motion of Sb‐Cl bond, since such low frequencies can only come from the metal‐halogen bond vibrations; similar Raman bands in the range of 70 – 120 cm^−1^ with Pb–I bond vibrations were reported and probed via DFT studies.^[^ ^45^ ^]^

By fitting to the peaks of the Raman spectra in Figure 5a, the frequencies and the spectral width were determined, and they are summarized in Figure 5b,c, respectively. Regarding Figure 5b, peaks A_1*g*_, E_*g*_, and T_2*g*_ are shifted to higher wavenumbers as the pressure is raised; this continuous shifting to higher frequencies of the A_1*g*_, E_*g*_, and T_2*g*_ modes is accounted for by the gradual shrink of the crystal lattice, with increasing the pressure. In addition, the broadening of peak A_1*g*_ (Figure 5c) suggests that the perovskite becomes slightly less crystallized as the pressure is increased.

Referring back to Figure 5b, however, the frequency of peak S is decreased as the pressure is increased from 1 atm to 4.8 GPa, and the peak becomes too weak to identify above 4.8 GPa. This disappearance of peak S above 4.8 GPa in Ramam spectrum is reminiscent of the observation in the PL spectrum (Figure 4) where peak S also appears too weak above 4.8 GPa, indicating a good correlation between the Raman spectrum and the PL spectrum. As discussed above, this Raman peak corresponds to the Sb‐Cl bond vibrations, so it is the [SbCl_5_]^2 −^ polyhedron that plays an important role as the PL emission center for peak S in Figure 4a. In addition, the evolution of the XRD pattern of the co‐doped sample is also presented in Figure S11 (Supporting Information), and there is no evidence regarding phase transition with increasing the pressure, even above 4.8 GPa. Note that this high‐pressure XRD experiments were performed with the X‐ray wavelength of 0.71 Å (Mo Kα); the diffraction angles are different from the XRD patterns at the ambient pressure (Figure 1b) that were obtained using the X‐ray wavelength of 1.54 Å (Cu Kα).

DFT calculations were also performed under various pressures. It is found that the structures are all similar, also indicating no evident phase change under pressure. The structure at 4.8 GPa is given in Figure S12 (Supporting Information) as an example. By carefully examining the Sb‐Cl bond lengths and corresponding bond angles (Table S4, Supporting Information), as the pressure is increased, all angles are further away from 90°, revealing that the [SbCl_5_]^2 −^ polyhedron is more distorted. Besides, most bond lengths are decreased with pressure, and this infers an overall contraction of the polyhedron. Interestingly, only the Sb‐Cl bond that is opposite to the Cl vacancy (Sb‐Cl4 bond in Figure S12, Supporting Information) is first decreased below 4.8 GPa, and is then increased above 4.8 GPa. This observation is well correlated to above PL and Raman experimental results, and this particular Sb‐Cl bond may be a factor that can influence the high‐pressure behavior of the perovskite.

Referring to Figure 5a again, the Raman spectrum is completely recovered after the pressure is released back to 1 atm, indicating that the phosphor synthesized in this work not only can withstand an ultra‐high pressure of 23.5 GPa, but it is also well reversible after the pressure is released. The high‐pressure experiments suggest the co‐doped phosphor has potential in ultra‐high‐pressure optoelectronic applications.

**Figure 6:**
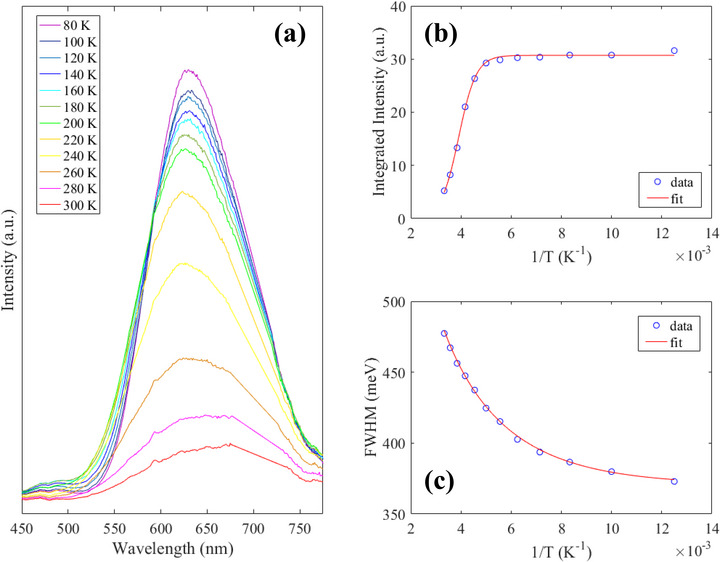
a) Temperature‐dependent fluorescence spectra of Cs_2_SnCl_6_:Sb^3 +^/Te^4 +^. b) Integrated PL intensity and c) FWHM, as a function of temperature.

To further probe the luminescent properties of the co‐doped phosphor, temperature‐dependent PL measurements were performed, and the spectra are shown in **Figure** **6**a. With raising the temperature from 80 to 300 K, the PL intensity is decreased, while the full width at half maximum (FWHM) is increased. This trend is known to the perovskite community that at high temperature the phonon‐exciton coupling that assists the non‐radiative recombination is strengthened, reducing the PL intensity and broadening the FWHM; similar effects were also reported and addressed in literatures^[^ ^46^, ^47^ ^]^. To quantify the phosphor's characteristics, the values of the integrated intensity and FWHM were determined from the PL spectra, and they are plotted in Figure 6b,c as a function of temperature, respectively.

Regarding Figure 6b, the exciton binding energy (*E*_*b*_) may be estimated by the Arrhenius law as following:^[^ ^31^ ^]^

(1) $$I\left(T\right)=\frac{I_{0}}{1+Cexp(-\frac{E_{b}}{k_{B}T})}$$

where *T* is the temperature, *I*_0_ is the emission intensity at 0 K, C is coefficient, and *k*_*B*_ is Boltzmann constant. By fitting the PL intensity to Equation (1), *E*_*b*_ was derived to 247 meV; the high value of the exciton binding energy indicates the stability of the STEs formed in the perovskite with the highly localized nature.

From Figure 6c, the electron–phonon interaction of the system can be described via the Huang–Rhys factor (*S*) and the phonon energy (ℏω_*phonon*_), and the temperature‐dependent FWHM is given by the following equation:^[^ ^31^ ^]^

(2) $$FWHM\left(T\right)=2.36\sqrt{S}\hbar \omega_{phonon}\sqrt{coth(\frac{\hbar \omega_{phonon}}{2k_{B}T})}$$

The best fit values are *S* = 16.1, and ℏω_*phonon*_ = 29.9 meV. The great value of the Huang–Rhys factor implies that there exists a strong electron–phonon coupling in the lattice of the phosphor. Besides, the value of the phonon energy is also similar to that was reported on another tin based perovskite (Ref. [31]).

## 5 Conclusion

Non‐toxic perovskites with outstanding luminescence properties are of significance in sustainable material community. Here, a novel lead‐free perovskite has been successfully synthesized, via isovalent and heterovalent co‐doping engineering. The color of the phosphor can readily be tuned due to the efficient energy transfer between STEs induced by the Sb and Te co‐dopants. The phosphor can withstand an ultra‐high pressure of 23.5 GPa, several interesting pressure‐dependent fluorescence properties have been revealed, and the underlying physics have been addressed. The synergy effect of the Sb and Te co‐doping has also been revealed, including the fast relaxation, narrow energy band, as well as the mixing of the EL spectra of the LEDs. The isovalent and heterovalent co‐doping chemical engineering scheme may promote future studies on other non‐toxic perovskites, and more appealing luminescence‐related applications may be found.

## Conflict of Interest

The authors declare no conflict of interest.

## Author Contributions

X.H. and T.X. contributed equally to this work. X.H. carried out the synthesis, investigation, analysis, and writing. T.X. performed the theoretical calculations and analysis. X.W. performed the high‐pressure experiments. Y.H. designed the experiments. Q.G. and N.S. carried out the XPS experiments. L.J. provided the VASP resources. C.S. designed the experiments, performed the investigation, analysis, and writing. Y. Z. carried out the high‐pressure analysis. H.L. provided the high‐pressure resources.

## Supporting information

Supporting Information

## Data Availability

The data that support the findings of this study are available from the corresponding author upon reasonable request.
