# Supplementary material for: Ultra‐High Pressure Phosphor Based on an Eco‐Friendly Perovskite Via Isovalent and Heterovalent Co‐Doping Engineering
Source: Adv Sci (Weinh). 2025 Nov 14;13(4):e15142. doi: 10.1002/advs.202515142 (PMC12822403; doi:10.1002/advs.202515142)
Supplement: Supplementary file 1 — Supporting Information [file ADVS-13-e15142-s001.pdf]

Supporting Information

Ultra-High Pressure Phosphor Based on an Eco-Friendly Perovskite via Isovalent and Heterovalent Co-Doping Engineering

Xinyu He[1,2,†], Tao Xiong[1,†], Xiyao Wang[3], Yongchang Han[2], Qingqin Ge[4], Nannan Shi[4], Liwei Jiang[5], Cheng Sun[1,*], Yinan Zhang[6,*], Hengnan Liang[6,*]

[1]*College of Physical Science and Technology, Dalian University, Dalian, 116622, China*

[2]*School of Physics, Dalian University of Technology, Dalian, 116024, China*

[3]*College of Science, Beihua University, Jilin, 132013, China*

[4]*Thermo Fisher Scientific China, Shanghai, 201203, China*

[5]*Key Laboratory of Physics and Technology for Advanced Batteries (Ministry of Education), College of Physics, Jilin University, Changchun, 130012, China*

[6]*College of Physics, Jilin University, Changchun, 130012, China*

*Corresponding authors: Cheng Sun, Yinan Zhang and Hengnan Liang

E-mail address: suncheng@dlu.edu.cn; zhangyinan@jlu.edu.cn; lhn@jlu.edu.cn.

[†]Xinyu He and Tao Xiong contributed equally to this work.

Materials

Cesium chloride (CsCl, 99%, Aladdin), tin chloride ($SnCl_2$, Alfa Aesar, 99.999%), antimony chloride ($SbCl_3$, 99%, Aladdin), tellurium chloride ($TeCl_4$, 99% ,Macklin), and hydrochloric acid (HCl, 37 wt% in water, Tianjin Kemi Ou Chemical Reagent Co., Ltd., China) were the starting reagents. All chemicals were used without further purification, unless otherwise stated.

Synthesis of Sb & Te Co-Doped Cs_2SnCl_6

A total of 2 mmol CsCl, 0.8 mmol $SnCl_2$, 0.1 mmol $SbCl_3$, and 0.1 mmol $TeCl_4$ powders were added into a 25 mL polytetrafluoroethylene (PTFE) container, with 5 mL of 37% hydrochloric acid. The container was placed into a muffle furnace. The mixture was heated at 180°C for 10 h. Crystals were obtained by slowly cooling the solution down to room temperature over the course of 30 h. Crystals of Cs_2SnCl_6:Sb^{3+}/Te^{4+} were separated by immediate filtration and washed with methanol. The product was dried in a vacuum oven overnight at 60°C.

The products were collected from the precursor solution by filtration followed by methanol rinse for three times. It was believed that this thorough wash was enough to remove the surface-adsorbed Sb and Te ions, and the detected Sb and Te discussed in this work should exist as Sb and Te dopants in our products. The molar ratios of the starting materials were chosen to be Sb/(Sn+Sb+Te) = Te/(Sn+Sb+Te) = 10%.

Fabrication of LED Devices

The LED devices were prepared by combining UVLED chips (370nm) with the synthesized $Cs_2SnCl_6:Sb^{3+}$, $Cs_2SnCl_6:/Te^{4+}$ or $Cs_2SnCl_6:Sb^{3+}/Te^{4+}$ phosphors. The prepared phosphor was first mixed with epoxy resin thoroughly. The bubbles in the silicone were then removed through a vacuum chamber and the mixture was applied to the LED chip, and finally cured at 80°C for 2 h to manufacture the LED devices. The current that was applied for the electroluminescence measurements was 40 mA.

Theoretical Calculations

Density functional theory (DFT) calculations were carried out using the Vienna ab initio simulation package (VASP). For expanding electronic wave functions, the cutoff energy of the plane-wave basis was set as 400 eV, and the Γ-only *k*-mesh was used to sample the Brillouin zone. The Cs_2SnCl_6 system was built by a 2 × 2 × 2 supercell of 288 atoms. Furthermore, the $Cs_2SnCl_6:Sb^{3+}$ system was modeled with one Sn atom substituted by one Sb atom, creating a Sb^{3+} doping situation. Other doping systems were also modeled using the similar method. The generalized gradient approximation (GGA) with PerdewBurkeErnzerhof (PBE) functional was employed to describe exchange-correlation interactions. The projector-augmented wave (PAW) pseudopotentials were used to describe the electron-ion interactions. The energy convergence for electron densities was limited to 1×10^{-5} eV and the geometry was considered converged when the force on every relaxed atom was below 0.03 eV $Å^{-1}$.

Measurements and Characterizations

The steady-state photoluminescence (PL) spectra and photoluminescence excitation (PLE) spectra were measured on the picosecond time-resolved fluorescence spectrometer (Pico-1000, Dalian institute of chemical physics).

The time-resolved PL measurements (TRPL) were excited by frequency-adjustable picosecond laser pulses at 310 nm and 360 nm, respectively, collected by the Pico-1000 spectrometer with laser.

X-ray diffraction (XRD) analysis were obtained by using the Rigaku Smart Lab 9 kW X-ray Powder diffractometer (Tokyo) operator, with the X-ray wavelength of 1.54 Å (Cu Kα).

High-pressure XRD experiments were carried out via the Rigaku Nanopix operator, with the X-ray wavelength of 0.71 Å (Mo Kα).

Scanning electron microscopy (SEM) and energy-dispersive (EDS) X-ray spectroscopy were performed with a SU-3500 scanning electron microscope.

Transmission electron microscope (TEM) and high resolution TEM (HRTEM) were recorded with a JEM-F200 transmission electron microscope at an accelerating voltage of 200 kV.

X-ray photoelectron spectroscopy (XPS) was carried out with a Thermo Fisher Scientific Nexsa.

The transient absorption spectra (TAS) were measured on an NTAS LFP1000 system in air at room temperature with an external 532 nm laser.

The in-situ high pressure PL was excited with a 355 nm semiconductor laser with an output power of 10 mV, and the spectra were collected by an optical fiber spectrometer (Ocean Optics, QW65000).

A specialized high-pressure Raman spectroscopy system was implemented using an SR-500i spectrometer (ANDOR) with 532 nm laser excitation.

Temperature-dependent PL spectroscopy measurements excited at 350 nm were carried out by a Quanta Master 8000 spectrometer.

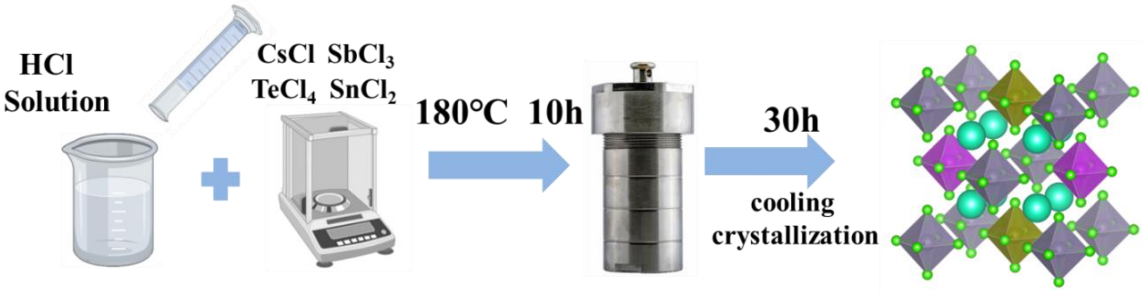

Fig. S1. Schematic illustration of the synthetic process.

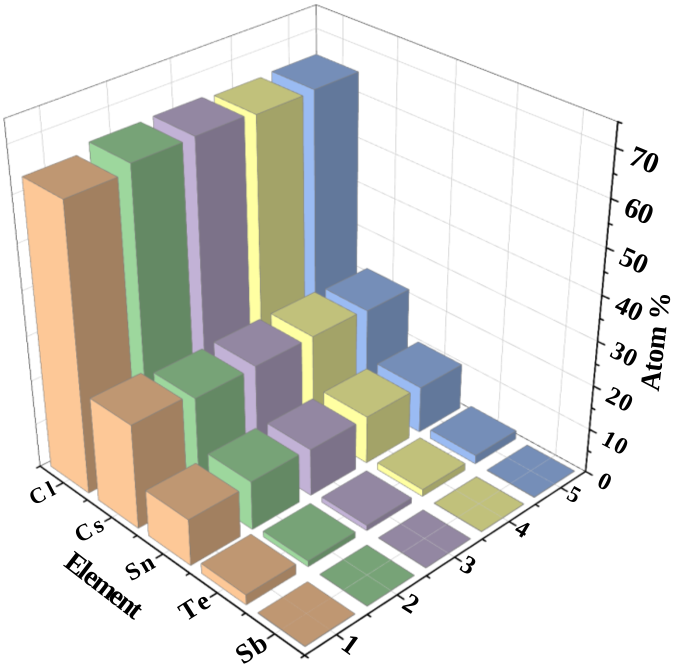

Fig.S2. Elemental distribution histograms across 5 different grains, determined from EDS analysis of $Cs_2SnCl_6:Sb^{3+}/Te^{4+}$.

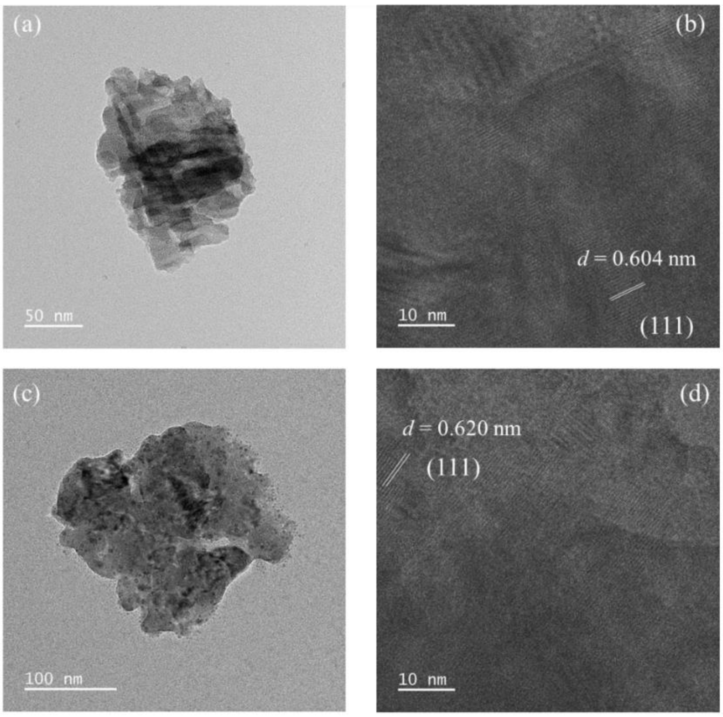

Fig.S3. TEM images of (a) Cs_2SnCl_6, and (c) $Cs_2SnCl_6:Sb^{3+}/Te^{4+}$. HRTEM patterns of (b) Cs_2SnCl_6, and (d) $Cs_2SnCl_6:Sb^{3+}/Te^{4+}$. The values of *d*-spacings of (111) plane are indicated in (b) and (d), respectively. Note that the scales in (a) and (c) are different.

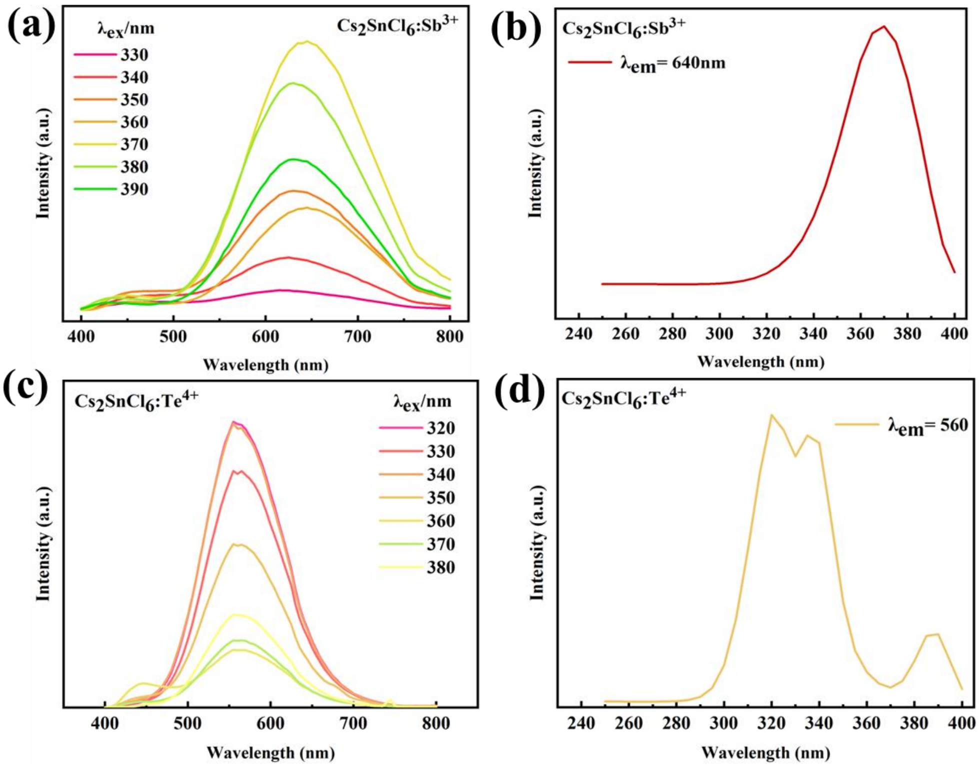

Fig. S4. (a) PL and (b) PLE spectra of $Cs_2SnCl_6:Sb^{3+}$. (c) PL and (d) PLE spectra of $Cs_2SnCl_6:Te^{4+}$.

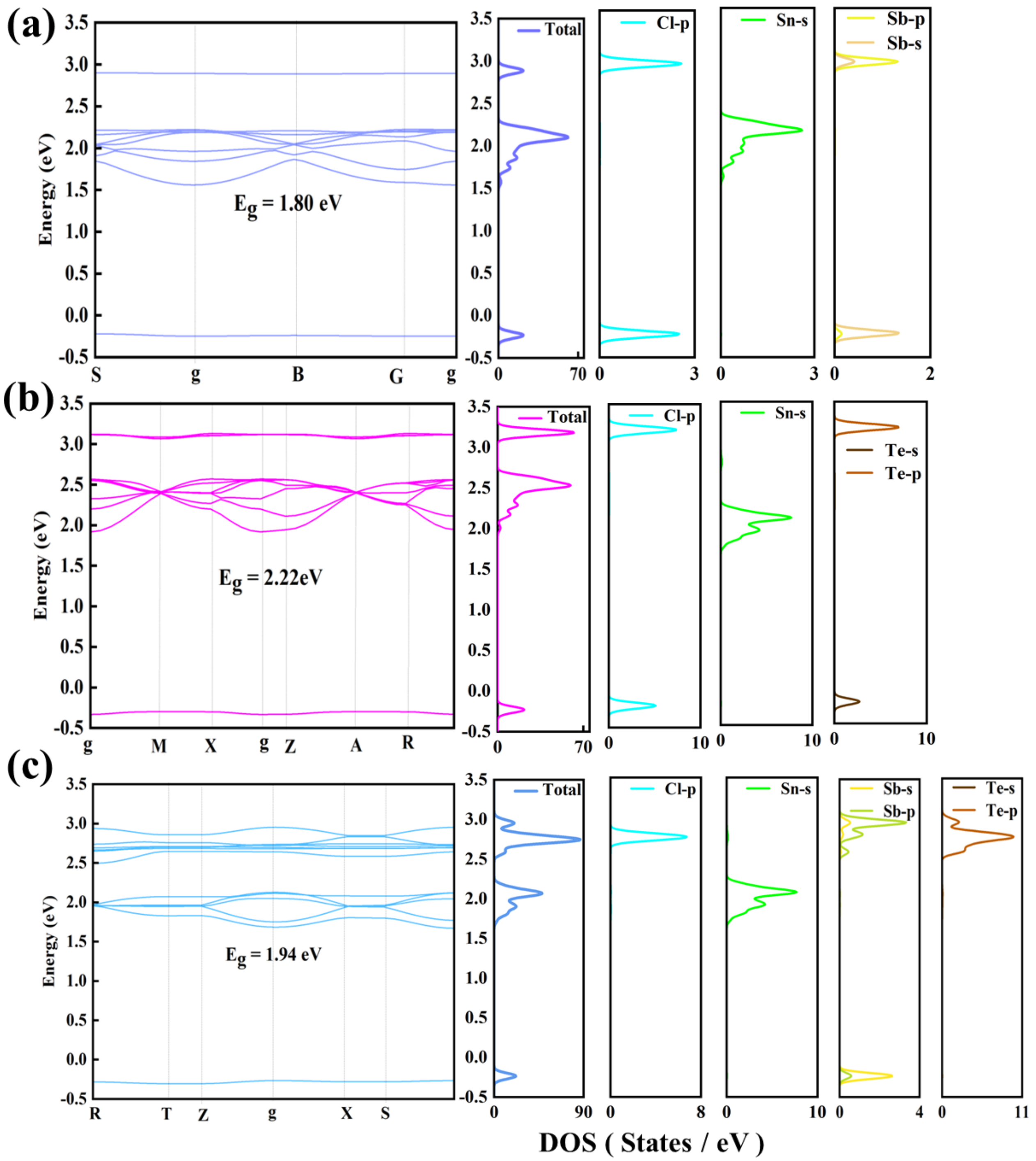

Fig.S5. Band structure and DOS of (a) $Cs_2SnCl_6:Sb^{3+}$, (b) $Cs_2SnCl_6:Te^{4+}$, and (c) $Cs_2SnCl_6:Sb^{3+}/Te^{4+}$.

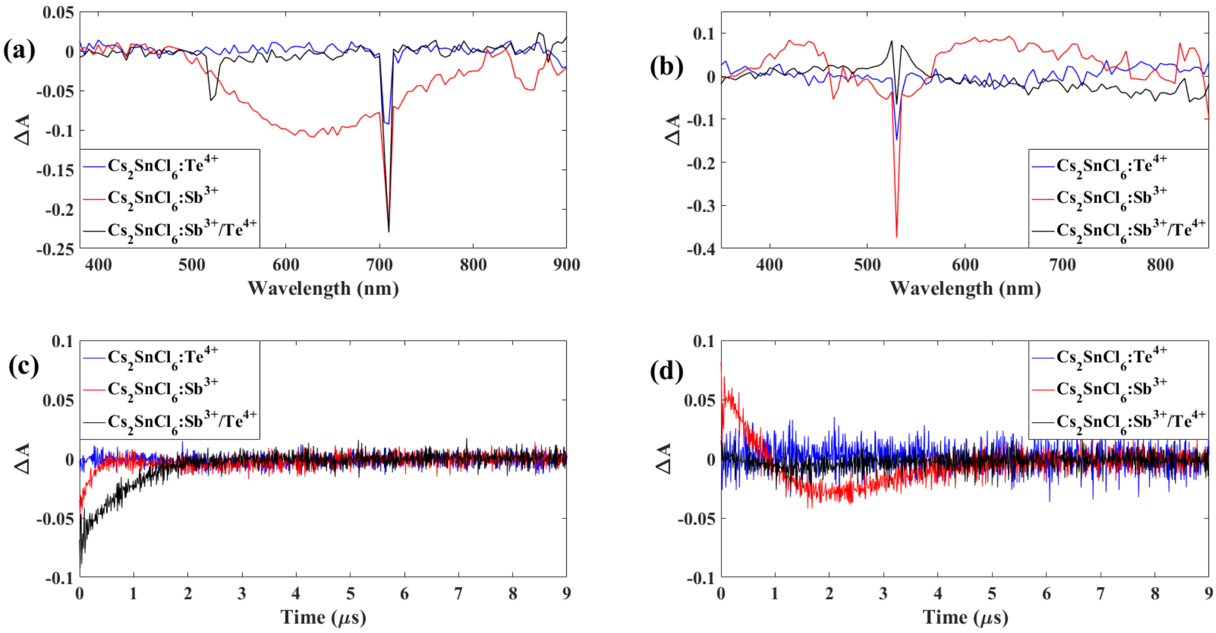

Fig.S6. PIA signals (ΔA) of Cs_2SnCl_6 with different dopants, upon photoexcitation (a) and (c) at 355 nm, and (b) and (d) at 532 nm. (a-b) ΔA as a function of wavelength, when the delay time was 0.06 μs. (c) ΔA as a function of time, when the probe wavelength was 520 nm. (d) ΔA as a function of time, when the probe wavelength was 435 nm.

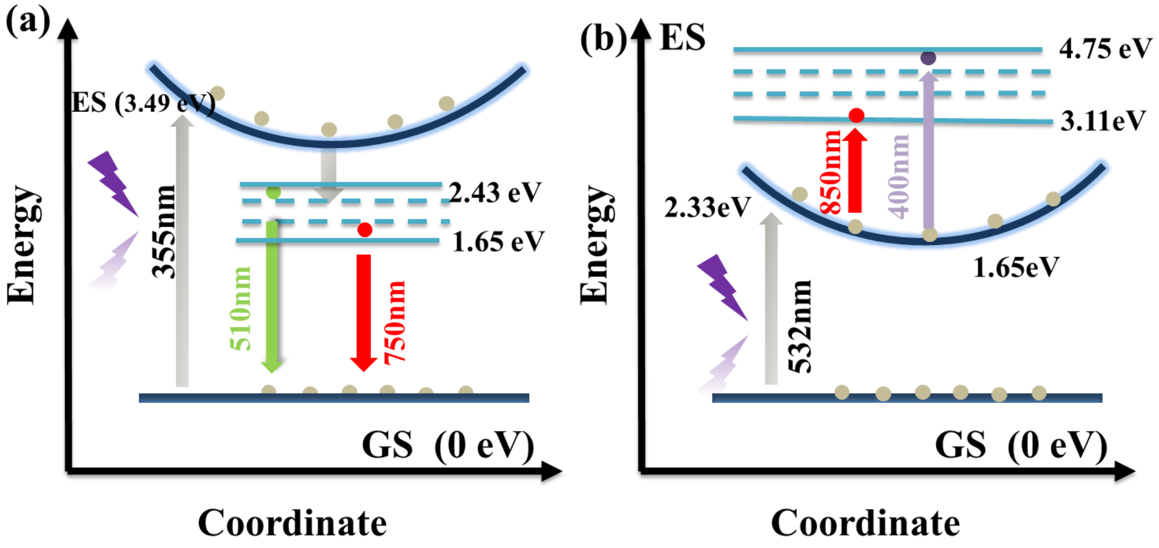

Fig. S7. Energy diagram for TAS dynamics of $Cs_2SnCl_6:Sb^{3+}$ excited at (a) 355nm, and (b) 532nm.

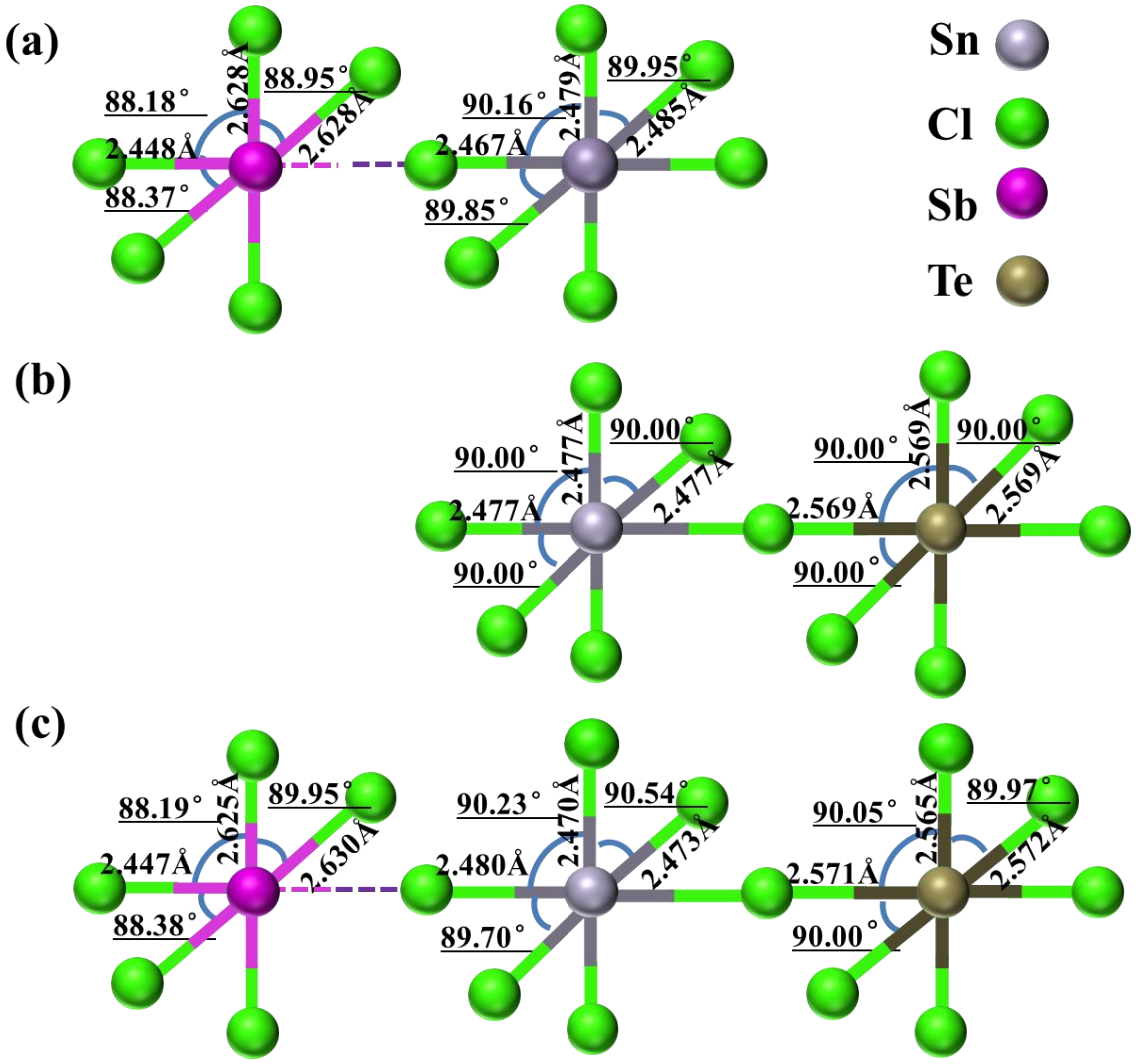

Fig.S8. Schematic diagram of the DFT-calculated crystal structure of (a) $Cs_2SnCl_6:Sb^{3+}$, (b) $Cs_2SnCl_6:Te^{4+}$, and (c) $Cs_2SnCl_6:Sb^{3+}/Te^{4+}$.

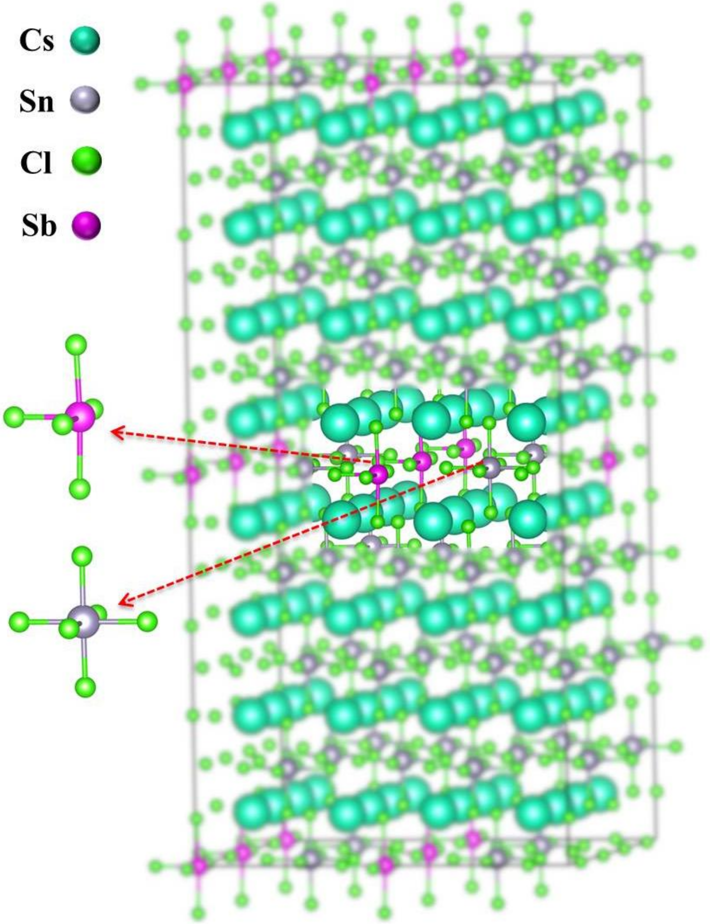

Fig.S9. DFT-calculated crystal structure of Cs_2SnCl_6:Sb^{3+}.

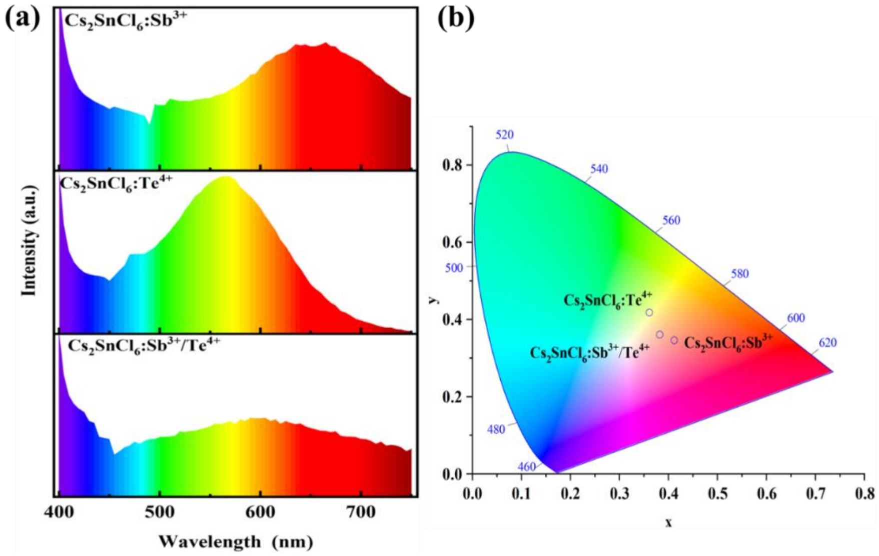

Fig.S10. (a) Electroluminescence spectra of the fabricated LEDs based on Cs_2SnCl_6 with different dopants. (b) CIE color coordinates corresponding to the fabricated LEDs: Cs_2SnCl_6:Sb^{3+} (0.41, 0.33), Cs_2SnCl_6:Te^{4+} (0.35, 0.41), and Cs_2SnCl_6:Sb^{3+}/Te^{4+} (0.37, 0.35).

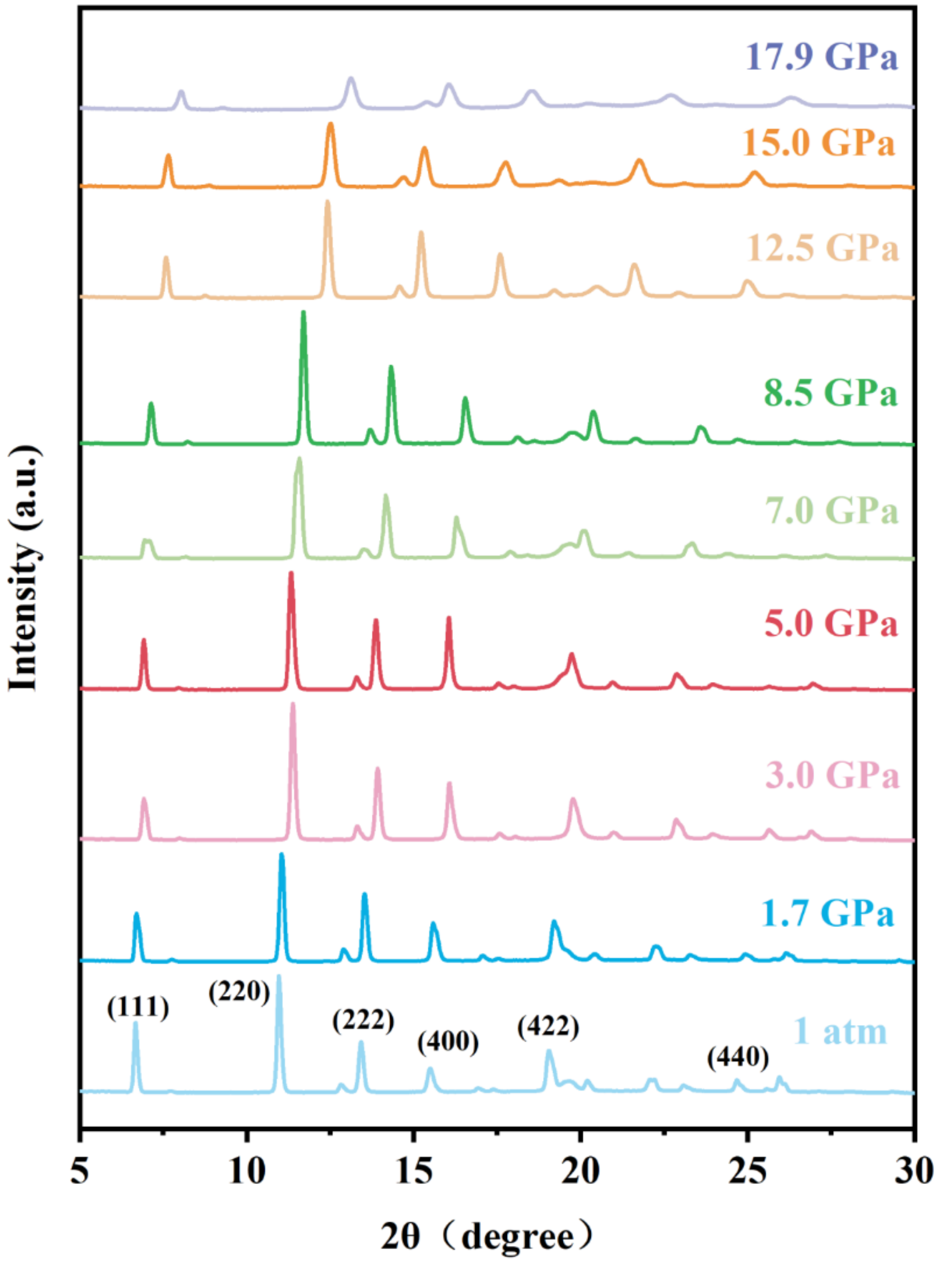

Fig.S11. XRD pattern evolution of $Cs_2SnCl_6:Sb^{3+}/Te^{4+}$ under pressure. Note that these high-pressure XRD experiments were performed with the X-ray wavelength of 0.71 Å (Mo Kα).

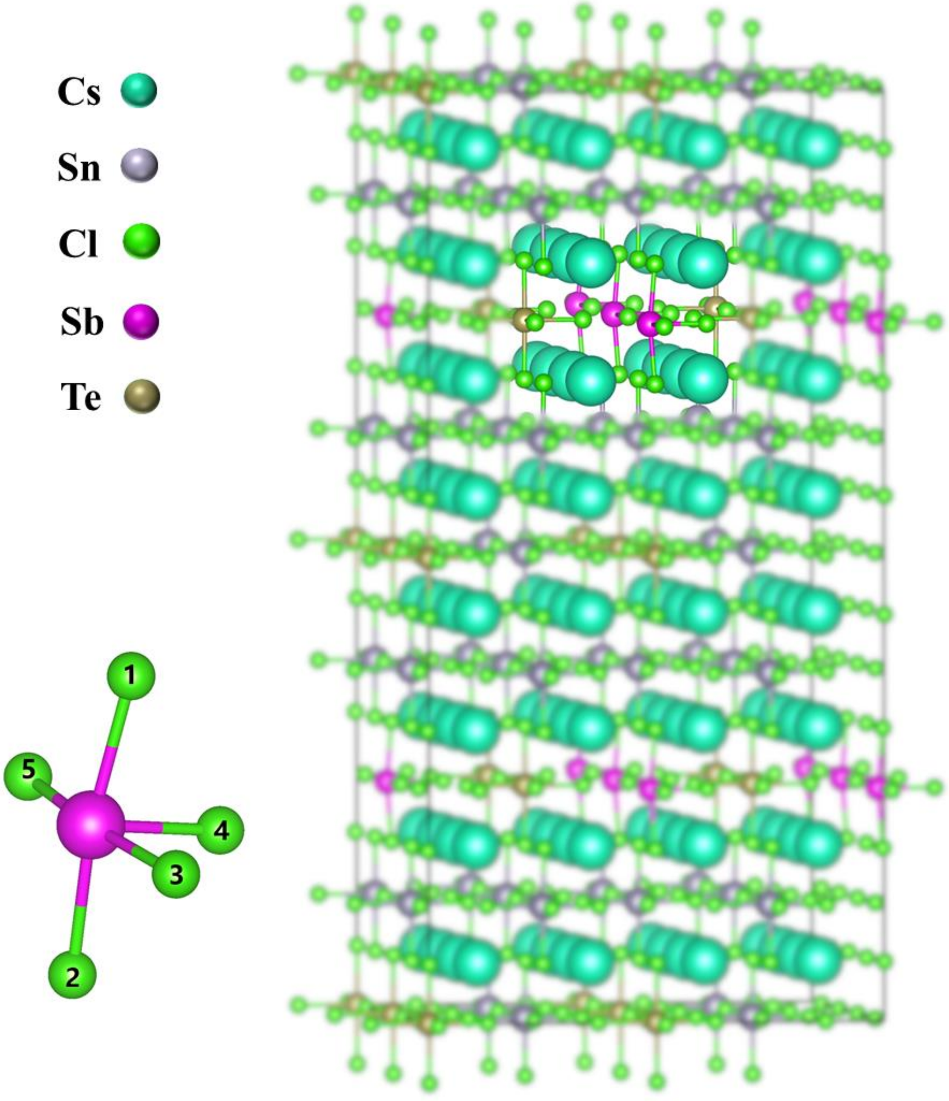

Fig.S12. DFT-calculated crystal structure of $Cs_2SnCl_6:Sb^{3+}/Te^{4+}$ under the pressure of 4.8 GPa.

Table S1. Atomic percentages for all elements of Cs_2SnCl_6:Sb^{3+}/Te^{4+} across 5 different grains, determined by EDS analysis.

Element (%)	1	2	3	4	5
Cl	63.67	65.36	65.54	64.96	65.29
Cs	23.45	22.16	22.40	22.43	21.93
Sn	10.46	11.05	10.72	10.91	10.67
Te	2.30	1.34	1.24	1.61	2.00
Sb	0.12	0.09	0.10	0.09	0.11
Total quantity	100.00	100.00	100.00	100.00	100.00

Table S2. Binding energy of each element in Cs_2SnCl_6 with different dopants.

Binding Energy (eV)	Cs^+ ($3d_{5/2}$)	Sn^{4+}($3d_{5/2}$)	Cl^-($2p_{3/2}$)	Sb^{3+}($3d_{5/2}$)	Te^{4+}($3d_{5/2}$)
Cs_2SnCl_6:Sb^{3+}	724.33	487.69	198.75	530.57	N/A
Cs_2SnCl_6:Te^{4+}	724.35	487.74	198.83	N/A	576.52
Cs_2SnCl_6:Sb^{3+}/Te^{4+}	724.33	487.69	198.81	530.22	576.00

Table S3. The values for the time constants (τ_α and τ_β), determined from the TRPL decay curves of Cs_2SnCl_6 with different dopants.

λ_{ex}= 310 nm, λ_{em} = 560 nm	Cs_2SnCl_6:Sb^{3+}	Cs_2SnCl_6:Te^{4+}	Cs_2SnCl_6:Sb^{3+}/Te^{4+}
τ_α (ns)	1.18 ±0.01	1.11 ±0.02	1.13 ±0.01
τ_β (ns)	14.09 ±0.28	18.01 ±0.57	12.82 ±0.29
λ_{ex}= 360 nm, λ_{em} = 640 nm	Cs_2SnCl_6:Sb^{3+}	Cs_2SnCl_6:Te^{4+}	Cs_2SnCl_6:Sb^{3+}/Te^{4+}
τ_α (ns)	2.13 ±0.11	2.64 ±0.04	1.97 ±0.06
τ_β (ns)	43.23 ±7.21	36.05 ±6.18	23.03 ±2.74

Table S4. Bond angles and bond lengths of $[SbCl_5]^{2-}$ determined from the DFT-calculated crystal structure of Cs_2SnCl_6:Sb^{3+}/Te^{4+} under pressure. The number labels of the Cl atoms are corresponding to those indicated in Fig.S12.

Pressure	1 atm	3.7GPa	4.2GPa	4.5GPa	4.8GPa	5.3GPa	5.8GPa	6.5GPa
Bond angle								
∠Cl1SbCl5	89.95 °	89.72 °	89.69 °	89.66 °	89.63 °	89.59 °	89.50 °	89.47 °
∠Cl1SbCl3	89.95 °	89.72 °	89.69 °	89.66 °	89.63 °	89.59 °	89.50 °	89.47 °
∠Cl1SbCl4	88.38 °	86.02 °	85.85 °	85.57 °	85.47 °	85.20 °	84.82 °	84.54 °
∠Cl3SbCl4	88.19 °	85.89 °	85.73 °	85.35 °	85.19 °	85.07 °	84.42 °	84.40 °
∠Cl4SbCl5	88.19 °	85.89 °	85.73 °	85.35 °	85.19 °	85.07 °	84.42 °	84.40 °
Bond length								
Sb-Cl1	2.63005Å	2.58727Å	2.58291Å	2.57968Å	2.57785Å	2.57120Å	2.56599Å	2.56100Å
Sb-Cl2	2.63005Å	2.58727Å	2.58291Å	2.57968Å	2.57785Å	2.57120Å	2.56599Å	2.56100Å
Sb-Cl3	2.62526Å	2.58237Å	2.57811Å	2.57334Å	2.57238Å	2.56620Å	2.55951Å	2.55541Å
Sb-Cl4	2.44769Å	2.44554Å	2.44553Å	2.44550Å	2.44562Å	2.44702Å	2.44722Å	2.44745Å
Sb-Cl5	2.62526Å	2.58237Å	2.57811Å	2.57334Å	2.57238Å	2.56620Å	2.55951Å	2.55541Å
